# New Insights about Treg and Th17 Cells in HIV Infection and Disease Progression

**DOI:** 10.1155/2015/647916

**Published:** 2015-10-19

**Authors:** Jacqueline María Valverde-Villegas, Maria Cristina Cotta Matte, Rúbia Marília de Medeiros, José Artur Bogo Chies

**Affiliations:** Immunogenetics Laboratory, Genetics Department, Genetics and Molecular Biology Post-Graduate Program, Institute of Biosciences, Universidade Federal do Rio Grande do Sul, Avenida Bento Gonçalves 9500, Prédio 43323, 91501-970 Porto Alegre, RS, Brazil

## Abstract

Treg and Th17 cell subsets are characterized by the expression of specific transcriptional factors and chemokine receptor as well as by secretion of specific cytokine and chemokines. These subsets are important to the differentiation, expansion, homing capacity, and recruitment of several different immune cell populations to the site of infection. Whereas Treg cells maintain self-tolerance and control the activation and expansion of autoreactive CD4^+^ T effector cells through an anti-inflammatory response, Th17 cells, in an exacerbated unregulated proinflammatory response, can promote autoimmunity. Despite such apparently opposite functions, Th17 and Treg cells share common characteristics, and their differentiation pathways are interconnected. Recent studies have revealed quite intricate relations between Treg and Th17 cells in HIV infection and progression to AIDS. Considering Treg cells, different subsets were already investigated in the context of HIV infection, indicating a fluctuation in the total number and frequency throughout the disease course. This review focuses on the recent findings regarding the role of regulatory T and Th17 cells in the context of HIV infection, highlighting the importance of the balance between these two subsets on disease progression.

## 1. Introduction

One of the major hallmarks of HIV infection is the immune activation that prompt viral replication and CD4^+^ T cells loss with disease progression, also leading to an impaired immune competence and consequently to AIDS development. It is still discussed if the loss of immune competence is caused by persistent immune activation, by a suppression of immune cells proliferation or by both phenomena [1].

The CD4^+^ T cells exert a central role in immune response and represent the preferential target of HIV infection. The most extensive studied CD4^+^ T cells lineages so far are Th1 and Th2, albeit HIV research now focuses on the immune balance and function of other cellular immune subsets, such as regulatory T cells (Tregs), T helper 17 (Th17), T helper 9 (Th9), and T helper 22 (Th22), where Treg/Th17 cells balance a relevant target of these studies [2, 3]. Treg cells, characterized by Forkhead Box Protein 3 (FoxP3^+^) expression, represent an important subset that control the proliferation of different immune cell subsets [4]. Meanwhile, T helper 17 most remarkable characteristic is IL-17 production that drives the capacity to these cells to exert an important proinflammatory function against extracellular pathogens [5]. Also, it is known that both subset phenotypes (Treg and Th17) are characterized by specific transcriptional factors and chemokine receptor expressions as well as by secreting specific cytokines and chemokines. Together, all these factors are important to the differentiation, expansion, homing capacity, and immunological cell recruitment into the site of infection or to the injured tissue for restraining the inflammation and dissecting the fine balance between Th17/Treg cells [6, 7].

Natural history of HIV infection involves a variable time of progression to AIDS. HIV long-term nonprogressors (LTNP) are characterized by long periods (>10 years) of AIDS-free symptoms even without antiretroviral treatment and maintain low levels of viremia and elevated CD4^+^ T cells counts. In contrast, rapid progressor (RP) HIV-1 subjects succumb to AIDS after a few years of infection [8]. Elite controllers (EC) are a particular group of LTNP, because they show persistent undetectable viremia (<50 RNA copies/mL) without treatment, although they represent less than 1% of all HIV-positive population [9]. Recent studies have focused the attention to elucidate the mechanisms involved in the variability of AIDS progression. Several components including viral factors and the host genetic diversity (e.g., the CCR5Δ32 variant and specific HLAs alleles) were already described as important factors that modulate HIV infection [10]. Nevertheless little is known about the cellular immune mechanisms involved in HIV progression and their role in immune molecular signaling, homing regulation, and cell-cell interactions. A better knowledge about these mechanisms could provide additional pieces to the complex puzzle of HIV pathogenesis. This review will focus on the recent findings regarding the role of regulatory T and Th17 cells in the context of HIV infection, highlighting the importance of the balance between these two subsets on disease progression.

## 2. The Role of Treg Cells on HIV Infection

### 2.1. Regulatory T Cells: Features and Functions

Regulatory T cells constitute a specialized subpopulation of CD4^+^ T lymphocytes in the immune system that exerts pivotal roles on establishing and maintaining self-tolerance and immune homeostasis. These specific functions are derived from the regulation of different immune cells proliferation [11]. Based on this, it is expected that Treg cells may participate in the immune regulation in human autoimmune diseases, cancer, allograft rejections, and virus infection [12–15].

As a definition, Treg cells express high amounts of CD4, CD25 (IL-2R*α*) and low CD127 (IL-7*α*) levels on the cell surface, although the Forkhead Box Protein 3 (FoxP3) is characterized as the gold standard marker for natural Treg cells (nTregs or tTregs, from thymic-derived regulatory T cells). IL-2R*α* and FoxP3 expression (mediated by STAT5) are critical for Treg cells survival and suppressive function [14, 16]. The limitation to the use of FoxP3 as a marker for Treg is that viable cells cannot be isolated after intracellular staining. In addition, FoxP3 expression is not always indicative of a regulatory status within human CD4^+^ T cells. A suggested alternative is the combined identification of the cell surface markers CD25 and CD127 (CD25^high^, CD127^low/−^) [17, 18]. In recent years, several studies have proposed a consensus panel of the markers to Treg immunophenotyping (Table 1). Another studied marker, CD39 (an ectonucleotidase involved in the hydrolysis of extracellular ATP into adenosine), identifies a bulk of human T cell regulatory population associated with high FoxP3 expression and inhibits T cell proliferation and cytokine secretion [19–21].

The suppressive capacity of Treg cells is widely dependent and influenced by several factors, such as IL-2, inhibitory cytokines (IL-10, TGF-*β*, or IL-35), CD152 (CTL-associated antigen 4, CTLA-4), and GITR (glucocorticoid-induced tumor necrosis factor receptor) [21]. IL-2 and, in a lesser degree, IL-7 and IL-15 cytokines are required for the correct differentiation of tTreg cells and the survival of tTreg cells and peripherally Treg cells (pTregs). Also, TGF-*β* seems to be an important cytokine involved on pTreg cells differentiation and homeostasis, although IL-2 is also required for TGF-*β*-mediated induction of FoxP3 [22]. Since several cytokines play a pivotal role on Treg cells function and differentiation, recent studies are investigating and suggesting their use on different conditions. The administration of IL-2 has been associated to increase in circulating Treg cells number and activation [23, 24]. IL-7 did not affect Treg cells proliferation but suppresses Treg cells capacity* in vitro* and* in vivo*. Also, IL-7 exerts a synergistic effect through downmodulation of the ectoenzyme CD39, favoring Th17 conversion [25]. In addition, expression of the enzyme indoleamine 2,3-dioxygenase (IDO), a tryptophan-degrading enzyme, represents another mechanism for immunosuppressive function [26].

### 2.2. The Role of Treg Cells in HIV Infection and Progression to AIDS: Friend or Foe?

Persistent immune activation is considered a reliable predictor for HIV disease progression and may lead to erosion, depletion, and exhaustion of the CD4^+^ T cell repertoire [27]. One of the immune mechanisms capable of controlling the activation and expansion of immune cells is the suppressive function exerted by Treg cells [28]. The role of Treg cells on HIV infection is still inconclusive since these cells can be involved both in the promotion as well as in the prevention of disease progression. Some findings point to a beneficial effect through suppression of chronic immune activation and inhibition of activated CD4^+^ T cells and consequent control of viral replication. On the other hand, a detrimental role is observed since the inhibition of specific HIV immune response through suppressive potential can promote viral persistence at the host [29, 30].

Considering that Treg cells express on their surface the chemokine receptor 4 (CXCR4) and chemokine receptor 5 (CCR5) molecules, these cells can potentially be susceptible to HIV R5-tropic and X4-tropic infection [31]. Some studies reported that HIV-infected Treg cells have its function and phenotype profile altered; however, opposite results have already been described [31–33]. Recently, Angin et al. [34] successfully isolated and* in vitro* expanded CD4 regulatory T cells from (HIV-positive) subjects. Expansion of functional Treg cells from blood and lymphoid tissues of HIV-infected subjects allied with its preserved suppressive capacity possibly indicates that these cells are not intrinsically defective in the context of HIV infection [34].

However, another study demonstrated that HIV-1 infection disrupts Treg cells function and its genes expression [35]. Treg cells infected with HIV-1 seem to be less potent in suppressing autologous CD8^+^ and CD4^+^ cell proliferation as compared to uninfected Treg cells. This impairment on Treg cells function can lead to HIV-associated generalized immune activation and inflammation [35]. According to this, infection of Treg cells with HIV X4-tropic strain results in a decrease of FoxP3 expression and decreased suppressive capacity [36]. Also, reduction in the expression of IL-2R*α* in Treg cells was observed in HIV-infected subjects with high viral load. This alteration could result in reduced Treg cells capacity function in these individuals, considering that the homeostatic role of this cells depends on IL-2 and the expression of IL-2R*α* at the cell surface [37].

Treg cells seems to be a major contributor to the immune activation observed during chronic HIV infection, since a strong relationship between Treg cells depletion and CD4^+^ T cell activation was observed [38, 39]. It is important to carefully observe that, in chronic HIV infection, a gradual increase of Treg cells (in terms of percentage) and a decrease of its absolute numbers, during progression of the disease, have already been described [39–43]. The opposite results regarding Treg cells relative and absolute frequencies are related to the fact that these cells are preferentially preserved compared to conventional CD4^+^ T cells [30]. Moreover, it is important to point that the discrepancy observed about Treg cells frequency on HIV infection can be attributed, at least in part, to (i) different surface markers used to characterize/isolate Treg cells; (ii) differential clinical stages of HIV disease, (iii) differences on sample analysed (blood or lymphoid tissues); and (iv) Treg subpopulations.

Human Treg cells have been subdivided according to their activation state: CD45RA^+^ are defined as naïve Treg cells and CD45RO^+^ defined as effector/memory Tregs cells in humans. Different cell subsets were already investigated in the context of HIV infection. When approaching the relative frequency (percentage), an increase of memory Treg cells and a decrease of naive Treg cells were observed as CD4^+^ T cells decline. The level of HIV viremia inversely correlates with memory and naive Treg absolute cell numbers. In addition, immune activation was inversely correlated with lower memory and naive Treg absolute cells numbers [17]. A distinct Treg cells phenotype was already identified on HIV infection. These cells express HLA-G on their surface but do not express FoxP3 or CD25 and are distinct in their profile and function from the classical regulatory T cells. However, these Treg cells (HLA-G^+^) seem to be diminished in progressive HIV-1 infection and may contribute to immune overactivation during disease progression [44].

It is noteworthy that Treg cells have an important role in immune homeostasis, and different evidences indicate that these cell repertoires can be disrupted in HIV infection. A better understanding of the Treg cells repertoire frequency and function in HIV-infected subjects with different patterns of progression to AIDS may help to elucidate the mechanisms affected by such cells on HIV pathogenesis and consequently their future therapeutic use.

There is an increasing number of studies approaching the role of Treg cells on different HIV progression groups, although the results are still conflicting. For instance, some of them suggest that low immune activation contributes to a slower disease progression [45]. Chase et al. [46] observed that Treg cells frequency and function were preserved among elite suppressor subjects (elite controllers), which may be a mechanism to limit immune activation. In the same line, Jiao et al. [47] observed a decrease of Treg cells absolute counts during HIV disease progression in the typical progressors group but not in LTNP subjects. One of the main reasons for the differences in Treg cells loss among distinct clinical progression groups would be that Treg cells migrate to lymphoid tissues in the typical progressors, but not in LTNP, which may contribute to Treg cells preservation on this last group and elite controller group [48]. According to this, lower levels of FOXP3^+^, CTLA-4, and TGF-*β*, but not IL-10, were observed in the tonsils of HIV-infected subjects classified in the nonprogression group compared to HIV typical progressors [33], indicating that the accumulation of Treg cells within lymphoid tissues is a feature of chronic progression. More recently, it was shown that viremic slow progressors subject has lower Treg cells numbers associated with CD4^+^ T cell decreased proliferation and surprisingly mucosal T cell activation. In this study, the low Treg cells numbers in the rectal mucosa may contribute to immune activation although they may also support stronger anti-HIV immune responses and a preserved Treg/Th17 cells balance [45].

Although some studies support the evidence of preserved Treg cells frequency and function in slow progressors subjects, there is no consensus in the literature since no differences among Treg cells frequency of slow progressors compared to HIV-infection acute disease and seronegative individuals have been described. Gaardbo et al. [49] showed no alteration on Treg cells numbers among LTNP, EC, viremic controllers, typical progressors, and HIV-seronegative individuals both in blood and in lymphoid tissues. However, activated Treg cells were elevated in LTNP and elite controllers compared to typical progressors and HIV-seronegative controls, whereas resting Treg cells were diminished, suggesting an important role of different Treg cells subsets on HIV pathogenesis [49]. In this same direction, Brandt et al. [50] observed a lower frequency of Treg cells in EC compared to viremic individuals (HIV-seropositive HAART-naïve), and the frequency was correlated with T cell proliferating and activation.

### 2.3. Treg Cells in Animal Models: Investigating Treg Cells on SIV Infection

Similar to HIV infection, the exact mechanism of regulatory T cells function as well as its frequency during Simian Immunodeficiency Virus (SIV) infection is unclear. Li et al. [51] observed a higher absolute and relative number of Treg cells in Chinese* Rhesus macaques* in the early stages after SIV infection. No alteration on Treg cells suppressive capacity after infection was described. Estes et al. [52] observed an important regulatory response (mediated by FoxP3^+^ and TGF-*β* ^+^ cells) after SIV exposure that may be involved in immune suppression of antiviral response and favor viral persistence. Although the majority of studies evaluate peripheral blood, Treg cells accumulation in lymphoid tissues was also described [53]. In addition to this, Tregs cells can potentially influence disease progression since lower FoxP3 mRNA levels were observed in an SIV nonprogressors model when compared to SIV progressors [33].

A study performed by Pereira et al. [54] investigated the frequency of Treg cells on two animal models with distinct profiles of SIV progression: African primate* Sooty mangabeys* (SM) (that do not develop immunodeficiency or disease) and Asian* Rhesus macaques* (RM) (a disease progression model). A decrease in Treg cells numbers was observed in chronically SIV-infected RM compared to uninfected animals. In longitudinal analysis, the SIVmac239-infected RM showed a transient increased Treg cells frequency in the acute phase of infection [54]. After the acute phase, a progressive decrease in the frequency and number of Treg cells was observed and correlated with high viral load. Antiretroviral treatment promoted an increase in the frequency and absolute count of Treg cells. None of these differences was observed on the SM model [54]. Another strategy used to investigate the role of Treg cells in HIV infection was to block Treg cells with an anti-CTLA-4 blocking antibody. CTLA-4 blockage in chronically SIV-infected ART-treated macaques was associated with lower IDO and TGF-*β* levels, as well as decreased viral RNA levels in lymph nodes and an increased immune specific response, suggesting a potentially therapeutic approach on HIV treatment [55].

### 2.4. The Impact of Highly Active Antiretroviral Therapy (HAART) on Tregs

Highly active antiretroviral therapy can significantly influence Treg cells numbers in HIV-infected subjects, decreasing or even normalizing its frequency at similar numbers to that of healthy controls [56, 57]. Some studies report that lower Treg cells numbers were found in blood and lymphoid tissues of treated compared to untreated subjects [58, 59].

Additionally, it has been hypothesized that Treg cells may contribute to the complete success of the treatment since subjects that do not respond to HAART seem to show higher Treg cells numbers as compared to responders [58, 60, 61]. Gaardbo et al. [62] also demonstrated that subjects with suboptimal immunological recovery had higher percentages of Treg cells and activated Treg cells, as well as lower resting Treg cells frequency in blood. In this same direction, higher levels of Treg cells in blood and lymphoid tissues predict a higher immunological reconstitution in individuals with low CD4^+^ T cell counts [62]. In a study performed by Jiao et al. [47], HAART increased peripheral Treg cells counts and induced a decrease in the immune activation and CD8^+^ T cell apoptosis in complete responders but not in nonresponders subjects. In conclusion, considering the important role of Treg cells in the balance between immune activation and/or suppression during HIV progression as well as its influence on HAART response, these cells may be useful as therapeutic targets or for prognostic monitoring in the future.

## 3. The Role of Th17 on HIV Infection

### 3.1. Th17 Cells: Features and Functions

Subpopulations of Th17 T helper lymphocytes were recently described and characterized by its involvement in mucosal immune inflammatory response, being its major function to protect the host against extracellular bacterial and fungal infections [5]. Th17 cells can be found under homeostatic conditions, particularly in the lamina propria of the small intestine [63]. However, during infection or under inflammatory conditions, Th17 cells can be induced in other tissues. This cellular lineage is responsible for the release of several cytokines that will act in nearby cells, inducing the production of chemokines able to recruit neutrophils and macrophages to the site of infection [64]. Further, Th17 cells can induce the expression of antimicrobial peptides, as lipocalin-2, Reg3*γ*, *β*-defensins, and calprotectin [65].

Th17 human cells are characterized by the expression of the transcription factor RORc and by the surface markers CD161, IL-23R, CCR6, and CCR4 [66, 67]. Moreover, the expression of CCR5 seems to be tissue-specific, with Th17 cells in the peripheral blood being predominantly CCR5-negative although they are CCR5-positive at the gastrointestinal tract [68]. The induction of RORc is dependent on STAT3, preferentially activated by IL-6, IL-21, and IL-23 in the presence of low amounts of TGF-*β* [69, 70]. Additionally, a balance between IL-6 and TGF-*β* concentrations has a pivotal role in driving Th17 immune responses, as will be better discussed later [71, 72].

Stimulated Th17 effector cells express several proinflammatory cytokines, such as IL-17, IL-21, IL-22, and IL-26, and chemokines as CXCL-6, CXCL-7, CXCL-8, and CCL20 [73], which contribute to the expansion of the inflammatory response through cells recruitment and activation and induction of antimicrobial peptides production. IL-17 leads to inflammation through NF-*κβ* and MAPKs and the induction of genes that code for matrix metalloproteinases, growth factors, other proinflammatory cytokines, and chemokines that attract neutrophils [74].

A balance of proinflammatory and anti-inflammatory or suppressive cytokines in the cellular microenvironment seems to be determinant to the differentiation of the Th17 cells population in specific subsets: Th17 cells expressing both Th17-Th1 and Th17-Th2 surface markers were found in response to the presence of IL-6, IL17, and IL-1*β* and addition of IL-12 or IL-4, respectively [75]. Another subset, Th17-Treg cells, seems to involve a more complex signalling context [76].

### 3.2. The Role of Th17 Cells in HIV Infection and Progression to AIDS

Th17 cells are constitutively observed throughout the intestinal lamina propria and in gut-associated lymphoid tissues (GALT). Approximately 80–90% of the CD4^+^ T cells present in GALT are able to secrete IL-17 [66]. Furthermore, it is recognized that GALT is the main region for HIV replication and massive CD4^+^ T cells depletion in early infection is observed in this compartment [77]. Indeed, extreme permissiveness of Th17 cells to HIV-1 infection can be explained based on the fact that mucosal CD4^+^ T cells present a CD45RO^+^ memory phenotype and express CCR5 and/or CXCR4 [78]. Therefore, the loss of Th17 cells during the HIV infection affects the intestinal mucosal barrier as well as local innate and adaptive immune functions [78].

The presence of HIV-specific Th17 cells in HIV-infected individuals during early infection was already reported; however, this response was not detectable during chronic or nonprogressive stages of the infection disease [79]. Conversely, Brenchley et al. [80] demonstrated that, in HIV-infected and uninfected individuals, Th17 cells respond to bacterial and fungal antigens; nevertheless, Th17 cells response was not specific for viral antigens, including HIV. However many studies found that massive infection of CD4^+^ T cells in GALT is directly associated with inflammation of the mucosal tissues and a breakdown of the mucosal integrity, resulting in microbial translocation from the lumen of the gut into peripheral blood [81, 82].

As has been suggested by some authors, Th17 cells may have dual impact on HIV infection due to the functional capacity in the mucosal tissue. In the acute phase of infection, in an inflammatory environment, Th17 cells could promote cell migration to the gut and create conditions for viral replication [83–85]. Nevertheless, in the chronic phase of infection, the reduced number of Th17 cells in the gut has been associated with a decrease in mucosal restoration and increase of microbial translocation and immune hyperactivation, which would contribute to exacerbation of the infection [80, 86].

Initial studies evaluating Th17 populations in HIV infected subjects demonstrated that Th17 cells were depleted in the gut-associated lymphoid tissue [39, 87]. In two subsequent studies, Salgado et al. [88] and Ciccone et al. [89] evaluated the numbers of Th17 cells in LTNP and typical progressor subjects. They reported similar results, suggesting that the number of Th17 cells in LTNP is greater than in typical progressor subjects. Furthermore, Salgado et al. [88] also observed a negative correlation between plasma HIV-RNA levels and Th17 cell number and with CD4^+^ IL7R^+^ cell number: HIV infected with higher of viral load showed the lowest numbers of Th17 cells and IL7R^+^CD4^+^ cells. These authors suggest that increased numbers of Th17 cells in LTNP subjects could better preserve the immune response against bacterial infections. Thus, low microbial translocation could explain the reduced activation and slower progression of the disease in LTNP subjects. Supporting these results, Singh et al. [90] showed that extensive elimination of CD4^+^ T lymphocytes in the GALT in the early stages of HIV-1 infection affects the intestinal homeostasis and significantly decreases the effector and regulatory functions of Th17 cells.

### 3.3. Th17 Cells in Animal Models: Investigating Th17 Cells on SIV Infection

Since Th17 cells of the SIV host have the same phenotype and general functions of the human Th17 cells, these cellular lineages have been investigated in different animal models, as* Sooty mangabeys* (SM) (that do not develop immunodeficiency or disease) and Asian* Rhesus macaques* (RM) (a disease progression model). The Th17 cells studies in SM can be highlighted because, in spite of severe depletion of CD4^+^ T cells in the mucosal tissues during acute SIV infection, even in the face of high viral replication similar to infections by HIV-human and SIV-RM, they do not progress to AIDS [91].

Raffatellu et al. [92] showed the inability of SIV-infected macaques to assemble an inflammatory GALT response against* S. typhimurium* due to an overall CD4^+^ T cells depletion in this tissue. Also, a significant systemic spread* S. typhimurium* after the loss of Th17 cells was observed. Another important study, by Paiardini et al. [93], revealed that, after nonpathogenic SIV infection, SM are able to maintain or increase the levels of Th17 cytokines due to the recovery of CD4^+^ T cells supported by the bone marrow and that this recovery contributes to the resistance against progression to AIDS. Other studies identified significant differences in the mucosal barrier integrity in models of HIV and SIV infection [91]. According to Brenchley et al. [94], Th17 cells are preferentially depleted in the mucosa of HIV^+^ humans and SIV^+^ *Rhesus macaque* pathogenic infections, but these cells were preserved in SM-SIV infections.

Recent studies have correlated the expression of CCR6 in Th17 cells and preservation of the gut mucosal barrier. This fact can be highlighted by the maintenance of Th17 cells in the gut and the reduced microbial translocation in SIV-infected RM treated with IL-21, a key cytokine in the activation of Th17 response [95]. Also, there are in the human Th17 repertoire, especially prevalent in the GALT, cells expressing high levels of CCR5, which would be a target of a preferential and rapid depletion [96].

SIV replication in the infected RM is restricted by the size of the preexisting Th17 cells compartment: animals with a high representation of such cells in blood and in the intestinal tissue previously to infection experienced peak and set-point viral loads about one log unit lower than those with a lower representation of Th17 cells [97]. Reciprocally, treatment of macaques with IL-2 and G-CSF before infection led to the depletion of Th17 cells, reduction of the ratio between Th17 and Treg cells, and higher viral loads for 6 months after infection [97]. These results suggest that the host immune system pool previous to the infection has an influence on the disease course after infection and provides a new framework for understanding interindividual variation in response to HIV-infection.

### 3.4. The Impact of Highly Active Antiretroviral Therapy on Th17 Cells

In the HIV infection, Th17 cells seem to be preferentially depleted in the intestinal mucosa and to a lesser extent in peripheral blood [77]. In the acute phase, the low levels of CD4^+^ T cells can be restored with the viral load reduction mediated by HAART. Macal et al. [98] showed that the highest level of CD4^+^ T cells restoration during HAART correlates with a substantial increase in mucosal Th17 cells and a decrease in inflammation markers. However, it is unclear why HAART cannot restore Th17 cells in the intestinal mucosa of some individuals: this same study observed that in some HIV-infected subjects a low level of immune activation persists in GALT despite long-term therapy. A possible explanation is that as Th17 cells are highly susceptible to HIV infection, this subset would be depleted early in HIV infection, leading to nonrestoration of the Th17 cells in spite of HAART. On the other hand, there are evidences showing that the paucity of the Th17-lineage committed precursor cells coincides with the Th17 polarization deficit in HIV chronically infected on HAART individuals versus HIV-negative controls [99]. Therefore, it can be suggested that the initial exhaustion of the precursor Th17 cell subsets in early stages, in some HIV-infected individuals, could be correlated with the Th17 restoration deficit despite an undetectable viral load. These studies are discussed in more detail further in this review (see What about the Balance between Th17 and Treg Cells in HIV Infection?).

Ndhlovu et al. [100] reported that healthy children exhibit a higher frequency of Th17 cells in the peripheral blood than HIV-infected children. Also, infected children with viral load greater than 50 copies/mL had a greater decrease in the frequency of these cells compared to children with undetectable viral load, suggesting that a preservation of Th17 cells depends on viral suppression [100]. Recently Pilakka-Kanthikeel et al. [101] comparing virologic responders and virologic failures HIV-infected children to uninfected pediatric subjects showed that microbial translocation persisted after 44 weeks in both responders and failures HIV-infected groups. A study by Alvarez et al. [102] performed* in vitro* demonstrated that virus replication can be suppressed by 3TC therapy, but the restoration of Th17 response observed in noninfected controllers was only achieved with the combination of 3TC and a “cocktail” of Th17 cytokines (IL-6, IL-1*β*, TGF-*β*, and IL-23). Taking into consideration that it was possible to restore Th17 response, it will be interesting to conduct more studies with such potential therapy.

## 4. Th17 and Treg Balance

The Th17/Treg balance is defined as “a state of equilibrium of the immune system that permits accurate and rapid protective responses against pathogens but curtails potential for causing harm to the host through targeting of ‘self' and provoking overexuberant inflammatory processes” [6]. It is known that Th17 and Treg cells have opposite roles in the development and outcomes of autoimmune/inflammatory diseases. Whereas Th17 cells can promote autoimmunity due to a proinflammatory response, Treg cells maintain self-tolerance and controls activation and expansion of autoreactive CD4^+^ T effector cells through an anti-inflammatory response [7]. However, Th17 and Treg cells share common characteristics, and their differentiation pathways are interconnected.

Recent reports demonstrated that Treg and Th17 cells have a high grade of plasticity due the fact that their initial differentiation is not an endpoint of T cell development [75, 76]. This plasticity allows a functional adaptation to various physiological situations during an immune response and might also be a critical disturbing factor for the Th17/Treg balance, leading to the immunopathogenesis of autoimmune/inflammatory diseases [75].

The maintenance of a Th17/Treg balance mainly depends on environmental factors and genetic predisposition. Besides, the plasticity of both cell subsets is highly dependent on the cytokine milieu and in the inflammatory context. Importantly, the commensal microbiota composition has a particularly significant influence in the immune system regulation and an imbalance in the gut microbiome could lead to alterations of immune responses in both GALT and periphery [6]. Of note, there are mechanisms of peripheral tolerance, achieved in large part through the action of Treg cells.

TGF-*β* is a critical factor for both Th17 and Treg cells, essential for inducing both RORc and FoxP3 [72, 103]. CD4^+^FoxP3^+^RORc^+^ cells represent a transient population, able to give rising to either Th17 or Treg cells depending on the local conditions. If sensing a proinflammatory environment, TGF-*β* induces RORc expression and Th17 cells differentiation [6, 104]. In the absence of an inflammation, TGF-*β* promotes FoxP3 expression and in combination with IL-2 promotes differentiation, expansion, and survival of Treg cells that maintain immune tolerance. This fact is due to a FoxP3-mediated inhibition of the activity of RORc and ROR*α*, resulting in abrogation of IL-17 and IL-23 expression [105].

It was observed that Treg cells can acquire a Th17-like phenotype. They are able to release IL-17 and express RORc and high levels of CCR6 but can retain a suppressive capacity (although this capacity is rapidly lost upon strong activation in the presence of IL-1*β* and IL-6) and FoxP3 expression (Th17Treg profile) [75]. Other studies focused on naïve cells as precursor population of Tregs and Th17 cells and observed that both subsets have a common precursor. It was observed that natural Tregs differentiate from CD25^+^ naïve T cells (NTregs) [106, 107]. Valmori et al. [108] reported that polarization of human Th17 cells preferentially occurs from FoxP3^+^ naïve Treg cells in the presence of IL-2 and IL-1*β* and is increased by IL-23 and TGF-*β*. Recently, Mercer et al. [109] named these Th17-like phenotype Treg cells as IL-17^+^ Tregs cells, due to the fact that this subset produces IL-17, and observed that naïve Treg cells (TNreg) expressing CCR6 have a predetermined capacity to differentiate into IL-17^+^ Treg cells with suppressive activity* in vitro*. They also observed that a small portion of naïve Treg cells expressing CCR6 have the propensity to polarize into Th17 cells. CCR6 is expressed by both Treg and Th17 cells and plays a significant role in Treg-mediated suppression and in the migration of Th17 cells to inflammatory sites [110].

Other important factors can influence the Th17/Treg balance. The fine-tuning of Treg cells upregulate chemokine and cytokine receptors in a pattern matching that of the immune T effector cells, whereas chemokine receptors such as CCR6 and CXCR3 facilitate the spatial proximity of suppressive Treg and inflammatory effector cells and cytokine receptors (e.g., IL-1R, IL-6R) that may compete for important factors, thus, limiting the activation or differentiation of T effector cells [6, 104].

Also, the stability of Treg cells has been questioned. It was observed that adoptive transfer of FoxP3^+^Treg cells into lymphopenic hosts leads to loss of FoxP3 expression in these cells and their differentiation into follicular T helper cells (Tfh) in Peyer's patches [111]. In contrast, in another study, CD25^+^CD4^+^ T cells were stable and did not lose FoxP3 upon adoptive transfer into lymphopenic hosts, whereas a relatively minor fraction of CD25^−^ or CD25^low^ FoxP3^+^ cells can lose FoxP3 expression and divert into effector T cell lineages [112].

Studies observed a reduction in Treg cell numbers and/or a loss of Treg function in animal models and human autoimmune diseases. Nevertheless, it is important to highlight that an increased number of Treg cells in autoimmune disease do not necessarily mean that these cells are able to control the immune response. As aforementioned, Treg cells have a certain degree of plasticity and can lose their suppressive function, especially under inflammatory conditions. Furthermore, data on peripheral Treg cell numbers and function in human autoimmune/inflammatory diseases are contradictory and remain subject to debate.

### 4.1. What about the Balance between Th17 and Treg Cells in HIV Infection?

Several studies were carried out to investigate the Th17/Treg balance state in typical progressors treated or untreated, EC, slow progressors, HIV-infected subjects, and SIV infection model [113]. Since Treg cells are developmentally linked to Th17 cells, the ratio of Th17 to Treg cells is used as an index of the relative balance between these two cell subsets. An impaired Th17/Treg balance in HIV-1 infection has a deleterious effect on gut mucosal immunity and fuels immune activation by enhancing microbial translocation [3, 80].

The importance of the Th17/Treg balance maintenance was highlighted by experiments performed in animal models using SIV infection. For instance, a loss of the Th17/Treg balance was observed in pathogenic SIV infection in Pigtailed Macaques (PTs) when compared with nonpathogenic infection in African Green Monkeys (AGMs). SIV-infected PTs, but not SIV-infected AGMs, rapidly developed systemic immune activation and a selective depletion of Th17 cells, suggesting that loss of the Th17/Treg balance is related to SIV disease progression [113].

Li et al. [114] observed a continuous loss of Th17 cells which was accompanied by a concomitant rise in the frequency of Treg cells, resulting in a Th17/Treg cells imbalance during the HIV-1 progression disease in untreated chronic HIV-1 infected followed up for more than 1 year. This study included a small group of EC and remarkably, Th17/Treg cells ratios in those elite controllers remained comparable with ratios observed for HIV-seronegative controls. Complementary to these data, in an Indian HIV-infected cohort, Th17 cells from peripheral blood were significantly more depleted in late stage infected as compared to early stage infected and slow progressor subjects. In this same study, Treg cells frequencies in the subjects with slow progression HIV-1 infection were comparable to the HIV-seronegative controls [115]. Another study performed by Brandt et al. [50] also observed that Th17/Treg cells ratio was similar in EC and HIV-seronegative controls. Taken together, these results suggest that the maintenance of the equilibrium between Th17 and Treg cells would correlate with a “better prognosis” in terms of disease course. In addition, in this study, in untreated viremic and treated HIV-infected subjects, the Th17/Treg cells ratio was lower compared with HIV-seronegative controls. Interestingly, a study followed up HIV/AIDS subjects before and after HAART and observed that the Th17/Treg cells ratio was significantly decreased before treatment, while HAART partially normalized the Th17/Treg cells ratio [116], suggesting that the HAART treatment can restore the Th17/Treg cells balance.

It has been recently shown that IDO induced tryptophan (Trp) catabolism promotes T cell differentiation into Treg cells through FoxP3 overexpression and suppresses the expression of RORc and the generation of Th17 cells [26]. Also, enhanced IDO activity was associated with HIV disease progression, and such activity leads to a Th17/Treg imbalance in the peripheral blood [117]. This chronic activation by IDO pathway diminishes the host's capacity to generate Th17 cells affecting the mucosal immune barrier critically dependent upon Th17 cells [3]. However, a recent study observed that IDO induced Trp catabolism into kynurenine that induces a harmful effect on the Th17/Treg cells ratio that may subsequently contribute to enhanced microbial translocation during HIV-1 infection. Importantly, EC compared to ART successfully treated and healthy subjects displayed a distinctive Trp catabolism characterized by similar Kyn/Trp ratios and preserved IDO expression and Th17/Treg cells ratios [118]. Thereby, efforts to prevent an imbalance (or restore a balance) of Th17/Treg in HIV-infected individuals could be envisaged as a potential treatment alternative.

Studies are showing that precursor populations of Treg and Th17 cells are target of HIV infection, and this phenomenon perturbs the Treg and Th17 cell polarization and consequently the balancing of these subsets. Mercer et al. [109] observed that IL17^+^Treg cells (derived from naïve Treg cells) are selectively reduced in number in HIV-infected subjects with suppressed viral loads through HAART. Then, DaFonseca et al. [99] showed that a Th17 polarization is impaired and this deficit coincided with the paucity of CD25^high^CD127^−^FoxP3^+^ (naïve Tregs or nTregs) and CD25^high^CD127^+^FoxP3^−^ (called double positive) subset cells in chronically HIV-infected aviremic subjects under HAART. In this study, the nTreg cells from recently infected untreated viremic subjects harbored higher levels of integrated/unintegrated HIV-DNA when compared with the same cells from chronically HIV-infected aviremic subjects under HAART. Finally, both recent studies suggest the requirement for new therapeutic strategies designed to the preservation of IL17^+^Tregs- and Th17-lineage committed naïve precursors.

## 5. Role of Chemokine Receptors on Th17 and Treg Cells: Implications for HIV Pathogenesis

### 5.1. Characterization of Th17 Cells by Chemokine Receptors and HIV Infection

Chemokine receptors have an important role in the phenotypic characterization of memory T cell subsets with distinct migration capacities and effector functions. The ligands for these receptors are inflammatory chemokines and chemoattractants, which are expressed in inflamed tissues and mediate the selective recruitment of different types of effector cells [119].

Memory CD4^+^ T cells are highly heterogeneous in its potential homing and effector functions against a specific pathogen. Studies associate the commitment of lineage and antigenic specificity of CD4^+^ T memory subsets with chemokine receptors expression [120–122]. It was observed that CCR4^+^CCR6^+^CD4^+^ T cells subsets produce IL-17 and express the transcription factor ROR*γτ* (Th17 profile) and were specific for* Candida albicans*, whilst CXCR3^+^CCR6^+^CD4^+^ T cells subsets produce IL-17 and IFN-*γ* and express the transcription factors ROR*γτ* and T-bet (Th1Th17 profile) and were specific for* Mycobacterium tuberculosis* [120].

There is emerging interest in the knowledge of the phenotype of HIV-infected CD4^+^ T cells, with several studies demonstrating that HIV is very selective in choosing its cellular targets. It is well established that memory CD4^+^ T cells are more permissive to HIV compared with naive T cells [77]. Thereby, it was observed that CCR4^+^CCR6^+^ CD4^+^ T and CXCR3^+^CCR6^+^CD4^+^ T cell subsets in peripheral blood were highly permissive to replication of both R5 and X4 HIV strains. Interestingly, these CD4^+^ T cell subsets showed a persistent decline during chronic infection despite antiretroviral therapy [84, 122]. More recently, it was observed that* Tetanus toxoid* and* C. albicans* specific CD4^+^ T cells with a Th17 profile (and high expression of CCR6 and its CCL20 ligand) were permissive to HIV infection, whereas CMV-specific CD4^+^ T cells with a Th17 profile were highly resistant to both R5 and X4 HIV strains [121]. These results show a preferential infection of peripheral CCR6^+^CD4^+^ T cells by HIV and the importance of different CD4^+^ T cell subsets against specific opportunistic pathogens that are depleted at different rates [123].

Studies observed that memory and effector Th17 cells are present in a subset of CCR6^+^ cells in both peripheral blood and inflamed tissues and are preferential target to HIV-1 infection [124]. Even though Th17 cells express more than one trafficking receptor in a tissue-specific manner, CCR6 is the unique receptor that is uniformly expressed by all subsets of Th17 cells [125]. CCR6 is a gut homing chemokine receptor and has a critical role in cell migration into Peyer's patches of the distal small intestine where CCL20 (MIP-3*α*) is expressed [125]. During normal development and immune homeostasis, CCL20 selectively attract CCR6-expressing lymphocytes and DCs to the mucosal surfaces, organizing lymphoid tissues, such as Peyer's patches, mesenteric lymph nodes, and GALT [126]. Th17 cells subsets express CCR6 and produce CCL20. The production of CCL20 from Th17 cells is regulated similarly as IL-17 (induced by TGF-*β* along with IL-6) [126]. In contrast, an* in vitro* study observed that CCR6 expression on Th17 cells is coordinately regulated by TGF-*β* and IL-2. TGF-*β*, but not IL-6, was able to induce CCR6 on T cells; conversely, IL-2 effectively suppressed the expression of CCR6 on Th17 cells [72, 125]. Th17 cells, by producing CCL20, could also attract other Th17 cells via CCR6, meaning that the production of CCL20 can lead to further recruitment of other CCR6-expressing Th17 cells and sustained chronic inflammation [126].

**Figure 1:**
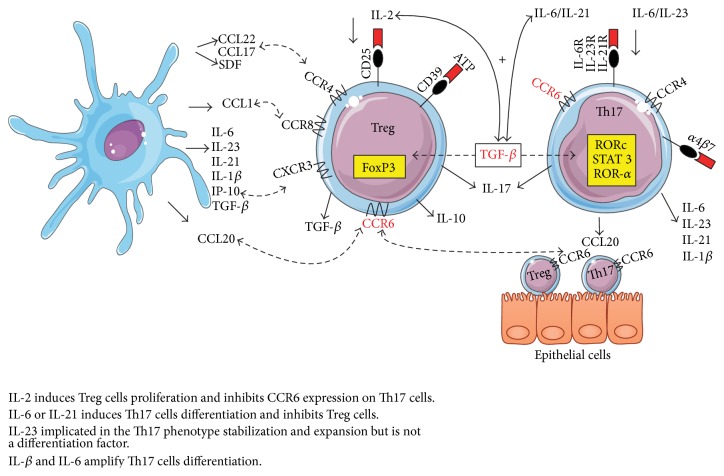
The interaction network between transcriptional factors, cytokines, chemokines, and their receptors in Th17 and Treg cells. The fine-tuning of Th17/Treg balance is regulated by expression of transcription factors that are activated by cytokines milieu and their receptors. TGF-*β* along with mainly IL-6 induces RORc, ROR-*α*, or STAT3 expression to differentiate Th17 cells while that in combination with IL-2 induces FoxP3 expression to differentiate Treg cells, while homing and immunological cells recruitment of both cell subsets are powerful mechanism mediated by chemokines and their chemokine receptors such as CCR6, CCR4, or CXCR3 which facilitates the recruitment of suppressive Treg and inflammatory effector Th17 cells (e.g., by means of CCR6-CCL20) into the site infection or injured tissue. Of note, other immunological cells, as dendritic cells, influence this balance because they produce cytokines, chemokines, and other molecules that participate in this interaction network.

The high susceptibility of Th17 cells to HIV* in vitro* is reflected by their* in vivo* depletion in the peripheral blood of HIV-infected individuals receiving treatment, compared with HIV-uninfected subjects [83]. It was suggested that CCR4^+^CCR6^+^ CD4^+^ and CXCR3^+^CCR6^+^ CD4^+^ cell subsets could have the potential to be recruited to the intestinal and vaginal mucosa through a CCR6^−^CCL20 dependent mechanism significantly contributing to HIV dissemination and persistence in cells, also attracting other CCR6^+^ CD4^+^ T cells to viral replication sites,* in vivo* [84, 122, 124]. Also, the *α*4*β*7 integrin identifies a subset of Th17 cells that is preferentially infected and depleted during acute SIV infection [125]. Accordingly, a study observed that the loss of peripheral *α*4^+^ *β*7^+^ memory CD4^+^ T cells correlates with the loss of CD4^+^ T cells in GALT during pathogenic SIV/HIV infection [127]. Taken together, these studies indicates that the ability of Th17 cells subsets to migrate into the GALT and other infection sites (e.g., periphery) depends on the imprinting for homing which is mediated by a combination of adhesion molecules and chemokine receptors (Figure 1).

### 5.2. Characterization of Tregs by Chemokine Receptors and HIV Infection

As aforementioned, homing and trafficking of effector cells are mainly facilitated by chemokines and expression of their chemokine receptors on distinct T cell subsets, and Treg cells are no exception. It was observed that CD45RA^−^ FoxP3^+^ T cells from peripheral blood express the CCR4, CCR5, CCR6, CXCR3, and CXCR6, chemokine receptors, which are commonly expressed by memory/effector T cells [128]. CCR4 and, even more, CCR8 have already been reported to be important for regulatory human CD25^+^CD4^+^ T cells [129]. Of note, mature dendritic cells preferentially attract Treg cells that express CCR4 and CCR8 through CCL17, CCL22, and CCL1 chemokine secretion (Figure 1). Thereby, it was suggested that CCR4 and/or CCR8 may guide Treg cells to inflamed areas and sites of antigen presentation in secondary lymphoid tissues in order to attenuate T cell activation or inhibit APC function [129]. This scenario suggests that chemokines secreted by APCs and chemokine receptors expressed on T cell subsets regulate the competition of T cells for access to antigen-bearing APCs.

Regarding CCR6, an important receptor expressed on Th17 cells, CCR6^+^ Treg cells exhibit a phenotype of activation, memory, and expansion that are typical for an effector memory function [110]. Unlike Th17, Treg cells do not produce CCL20 [126]. However, it was observed that Treg cells migrate towards to CCL20-producing Th17 cells* in vitro* in a completely CCR6 dependent manner (migratory response was completely abolished in CCR6-deficient Th17 and Treg cells) [126]. In this study, it is proposed that Th17 cells produce CCL20 that attract other CCR6^+^ Th17 cells as well as CCR6^+^ Tregs through CCR6.

In the context of viral infections, Qin et al. [130] observed that a simultaneous antagonism of CCR4 by increased CXCR3 ligand expression (CXCL11) and loss of CCR4 ligand expression contributed to reducing homing of FoxP3^+^ Treg cells to lymph node and intestinal tissues during SIV infection. In this study, the increment of IFN-*γ* as an upstream regulator of CXCR3 ligand expression and the decrease in TGF-*β* as an upstream regulator of IFN-*γ* expression revealed a complex set of interrelationship that control multiple positive and negative feedback system [130]. In the early stage of HIV infection, plasma IP-10 (CXCR3 ligand) levels were predictive of rapid progression than viremia or CD4^+^ T cells levels [131]. Regarding CCL20 (CCR6 ligand), saliva was shown to increase significantly CCL20 secretion. Thus, it suggests that saliva could facilitate HIV entry and other pathogens through the genital mucosa during sexual intercourse [132].

Nevertheless, little is currently known about how chemokines and chemokine receptors regulate the homing and trafficking of Treg cells in HIV infection. Differential profiles of Treg homing receptors could be critical in the control of the inflammatory response against HIV. Also, interactions between chemokines and their receptors, such as CCL20/CCR6-mediated signals, can be strongly induced by proinflammatory stimuli. Future studies approaching how Treg cell subsets interact with each other and with the remaining cells by means of their chemokine receptors would certainly help in the understanding of the HIV infection pathogenesis.

## 6. Conclusions

HIV-1 infection is characterized by a gradual decrease of the immunological competence and a massive depletion of CD4^+^ T cells, particularly in GALT, which leads to microbial translocation, contributing to immune hyperactivation, an important pathogenic mechanism HIV-1 infection. Th17 cells are proinflammatory CD4^+^ T cell subsets and play a pivotal role in host defense, mainly in the gastrointestinal tissue. Currently, most evidence suggests that Th17 cells have a beneficial role in HIV infection by promoting gut mucosa recovery, preventing microbial translocation and decreasing immune hyperactivation. However, a pathogenic role of these cells, particularly the induction of an increase in viral replication through the production of inflammatory cytokines, should not be ruled out. The role of Treg cells in regulating T cell activation during immune responses to pathogens such as HIV-1 is a subject of great interest. Their effects can be beneficial or detrimental depending on the balance between attenuating HIV-induced immune hyperactivation and mounting an immune response to HIV-1 and opportunistic pathogens.

The interaction between the cytokines milieu, chemokines, and chemokine receptors and the acquisition of tissue-specific homing form a complex network that is influenced mainly by the plasticity of T cells, genetic host, and environmental factors. Recent studies prompted that this network can disturb the Th17/Treg balance during HIV-1 infection. However, the mechanisms underlying this interaction are still not completely understood, and more studies need to be carried out in that direction. Finally, new findings about Th17/Treg outcomes and the understanding of interindividual variability in HIV infection will be crucial to the development of new treatment strategies and vaccines.

## Figures and Tables

**Table 1:** Immunophenotyping of Treg and Th17 cells and their precursors in different studies.

Subset cells	Markers used	References
Naive Tregs	CD45RA^+^CCR7^+^CD25^high^CD127^−^Foxp3^+^CD4^+^	DaFonseca et al. [99]
	CD45RA^+^CD45RO^−^CCR7^+^CD25^+^Foxp3^+^CD4^+^	Valmori et al. [107]
	CD45RA^+^CD25^+^CD127^low^Foxp3^+^CD4^+^	Valmori et al. [108]
	CD45RA^+^CD45RO^−^CD25^high^CD127^low^Foxp3^+^CD4^+^	Duhen et al. [18]
	CD45RO^−^CD25^+^CD127^low^CD4^+^	Tenorio et al. [17]

Memory Tregs	CD45RA^−^CD25^high^CD127^low^Foxp3^+^CD4^+^	Canavan et al. [133]
	CD45RA^−^CCR7^+/−^CD25^high^CD127^−^Foxp3^+^CD4^+^	DaFonseca et al. [99]
	CD45RO^+^CD25^high^CD127^low^Foxp3^+^CD4^+^	Duhen et al. [18]
	CD45RA^−^CD25^high^Foxp3^high^CD4^+^	Zhou et al. [134]
	CD45RO^+^CD25^+^CD127^low^CD4^+^	Tenorio et al. [17]

Memory Th17	CD45RA^−^CCR6^+^CCR4^+^CXCR3^−^CD4^+^	Gosselin et al. [122]; Becattini et al. [135]; Acosta-Rodriguez et al. [120]
	CD45RA^−^CCR6^+^CD26^+^CD161	DaFonseca et al. [99]
